# *N*′-*tert*-Butyl-5-(4-chloro­phen­yl)furan-2-carbohydrazide

**DOI:** 10.1107/S1600536808003486

**Published:** 2008-02-06

**Authors:** Xi-Chen Li, Ying Li, Zi-Ning Cui, Xin-Ling Yang, Yun Ling

**Affiliations:** a Department of Applied Chemistry, College of Science, China Agricultural University, Key Laboratory of Pesticide Chemistry and Application Technology, Ministry of Agriculture, Beijing 100094, People’s Republic of China

## Abstract

In the title mol­ecule, C_15_H_17_ClN_2_O_2_, the furan and benzene rings form a dihedral angle of 15.35 (8)°. In the crystal structure, inter­molecular N—H⋯O hydrogen bonds link the mol­ecules into chains extended in the [010] direction.

## Related literature

For general background, see: Wing (1988 ▶); Wing *et al.* (1988 ▶); Dhadialla *et al.* (1998 ▶); Heller *et al.* (1992 ▶); Mao *et al.* (2004 ▶). For details of some monoacyl­hydrazines and diacyl­hydrazines containing furan, see: Yang *et al.* (2002 ▶); Li *et al.* (2006 ▶).
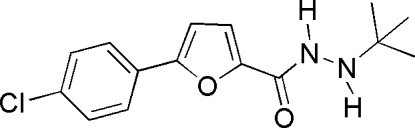


## Experimental

### 

#### Crystal data

C_15_H_17_ClN_2_O_2_
*M* *_r_* = 292.76
Orthorhombic, 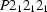

*a* = 9.3770 (7) Å
*b* = 9.7861 (7) Å
*c* = 16.0119 (12) Å
*V* = 1469.32 (19) Å^3^
*Z* = 4
Mo *K*α radiation
μ = 0.26 mm^−1^
*T* = 113 (2) K
0.32 × 0.24 × 0.20 mm

#### Data collection

Rigaku Saturn diffractometer
Absorption correction: multi-scan (*CrystalClear*; Rigaku/MSC, 2005 ▶) *T*_min_ = 0.921, *T*_max_ = 0.949
13814 measured reflections
3496 independent reflections
2754 reflections with *I* > 2σ(*I*)
*R*_int_ = 0.038

#### Refinement

*R*[*F* ^2^ > 2σ(*F* ^2^)] = 0.026
*wR*(*F* ^2^) = 0.056
*S* = 0.96
3496 reflections
192 parameters
H atoms treated by a mixture of independent and constrained refinement
Δρ_max_ = 0.22 e Å^−3^
Δρ_min_ = −0.21 e Å^−3^
Absolute structure: Flack (1983 ▶), 1490 Friedel pairs
Flack parameter: 0.00 (4)

### 

Data collection: *CrystalClear* (Rigaku/MSC, 2005 ▶); cell refinement: *CrystalClear*; data reduction: *CrystalClear*; program(s) used to solve structure: *SHELXS97* (Sheldrick, 2008 ▶); program(s) used to refine structure: *SHELXL97* (Sheldrick, 2008 ▶); molecular graphics: *SHELXTL* (Sheldrick, 2008 ▶); software used to prepare material for publication: *CrystalStructure* (Rigaku/MSC, 2005 ▶).

## Supplementary Material

Crystal structure: contains datablocks I, global. DOI: 10.1107/S1600536808003486/cv2380sup1.cif

Structure factors: contains datablocks I. DOI: 10.1107/S1600536808003486/cv2380Isup2.hkl

Additional supplementary materials:  crystallographic information; 3D view; checkCIF report

## Figures and Tables

**Table 1:** Hydrogen-bond geometry (Å, °)

*D*—H⋯*A*	*D*—H	H⋯*A*	*D*⋯*A*	*D*—H⋯*A*
N1—H1*A*⋯O2^i^	0.882 (15)	2.026 (16)	2.8744 (15)	160.9 (14)

Symmetry code: (i) 
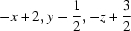
.

**Fig. 1.**
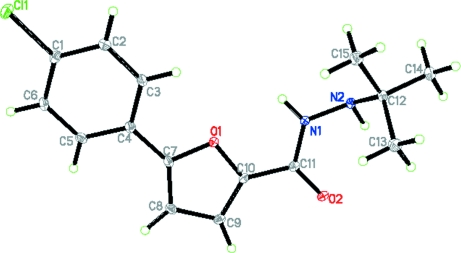
The molecular structure of (I), showing 50% probability displacement ellipsoids and the atom-numbering scheme.

## supplementary crystallographic information

### Comment

As one of molting hormone analogs, symmetrical *N'*-*tert*-butyl-*N,N'*-dibenzoylhydrazine (RH-5849) was first found to be a nonsteroidal ecdysone agonist in 1988 (Wing, 1988; Wing *et al.*, 1988). Afterward, several diacylhydrazine compounds were commercially developed as insect growth regulators (IGRs) which were widely used in agriculture (Dhadialla *et al.*, 1998; Heller *et al.*, 1992; Mao *et al.*, 2004). Recently, we synthesized a series of di- or mono- acylhydrazines containing furan for further study on the structure-activity relationship between monoacylhydrazines and diacylhydrazines. It was found that they both had good insecticidal activities (Yang *et al.*, 2002; Li *et al.*, 2006). In order to study the structural character and conformation of the monoacylhydrazine containing furan, the crystal structure of the title compound, (I), has been determined.

In (I) (Fig. 1), the benzene (C1—C6) and furan (O1/C7—C10) rings form a dihedral angle of 15.35 (8)°. The carbonyl group attached to the furan ring is almost coplanar with it. In the crystal, the intermolecular N—H···O hydrogen bonds (Table 1) link the molecules into chains extended in direction [010].

### Experimental

The title compound, (I), was synthesized by the reaction of 5-(4-chlorophenyl)furan-2-carbonyl chloride (0.96 g, 4 mmol) with *tert*-butylhydrazine hydrochloride (1.99 g, 16 mmol) using sodium hydroxide (10%, 8.0 g, 20 mmol) as the acid-binding agent. The mixture was stirred at room temperature for 5 h and filtered to obtain a yellow solution. Then the organic phase was separated and dried with anhydrous magnesium sulfate overnight. After removal of the solvent, the residue was purified by vacuum column chromatography on silica gel with petroleum ether and ethyl acetate as the eluent (V _petroleum ether_: V _ethyl acetate_ = 3:1) and then recrystallized from hexane–ethyl acetate (V_hexane_: V_ethyl acetate_= 1:1) to give colourless crystals suitable for X-ray diffraction (Li *et al.*, 2006).

### Refinement

Atoms H1A and H2A were located on a difference map and isotropically refined. The rest H atoms were positioned geometrically (C—H = 0.95–0.98 Å), and refined using a riding model, with *U*_iso_(H) = 1.2 or 1.5 *U*_eq_(C).

### Crystal data

**Table:**

C_15_H_17_ClN_2_O_2_	*D*_x_ = 1.323 Mg m^−^^3^
*M**_r_* = 292.76	Mo *K*α radiation λ = 0.71070 Å
Orthorhombic, *P*2_1_2_1_2_1_	Cell parameters from 3053 reflections
*a* = 9.3770 (7) Å	θ = 2.5–25.0º
*b* = 9.7861 (7) Å	µ = 0.26 mm^−^^1^
*c* = 16.0119 (12) Å	*T* = 113 (2) K
*V* = 1469.32 (19) Å^3^	Prism, colourless
*Z* = 4	0.32 × 0.24 × 0.20 mm
*F*_000_ = 616	

### Data collection

**Table:**

Rigaku Saturn diffractometer	3496 independent reflections
Radiation source: rotating anode	2754 reflections with *I* > 2σ(*I*)
Monochromator: confocal	*R*_int_ = 0.039
Detector resolution: 7.31 pixels mm^-1^	θ_max_ = 27.9º
*T* = 113(2) K	θ_min_ = 2.4º
ω scans	*h* = −12→12
Absorption correction: multi-scan(CrystalClear; Rigaku/MSC, 2005)	*k* = −12→12
*T*_min_ = 0.921, *T*_max_ = 0.949	*l* = −21→21
13814 measured reflections	

### Refinement

**Table:**

Refinement on *F*^2^	Hydrogen site location: inferred from neighbouring sites
Least-squares matrix: full	H atoms treated by a mixture of independent and constrained refinement
*R*[*F*^2^ > 2σ(*F*^2^)] = 0.027	*w* = 1/[σ^2^(*F*_o_^2^) + (0.0232*P*)^2^] where *P* = (*F*_o_^2^ + 2*F*_c_^2^)/3
*wR*(*F*^2^) = 0.056	(Δ/σ)_max_ = 0.001
*S* = 0.96	Δρ_max_ = 0.22 e Å^−^^3^
3496 reflections	Δρ_min_ = −0.21 e Å^−^^3^
192 parameters	Extinction correction: none
Primary atom site location: structure-invariant direct methods	Absolute structure: Flack (1983), 1490 Friedel pairs
Secondary atom site location: difference Fourier map	Flack parameter: 0.00 (4)

### Special details

**Table:**

Geometry. All e.s.d.'s (except the e.s.d. in the dihedral angle between two l.s. planes) are estimated using the full covariance matrix. The cell e.s.d.'s are taken into account individually in the estimation of e.s.d.'s in distances, angles and torsion angles; correlations between e.s.d.'s in cell parameters are only used when they are defined by crystal symmetry. An approximate (isotropic) treatment of cell e.s.d.'s is used for estimating e.s.d.'s involving l.s. planes.
Refinement. Refinement of *F*^2^ against ALL reflections. The weighted *R*-factor *wR* and goodness of fit *S* are based on *F*^2^, conventional *R*-factors *R* are based on *F*, with *F* set to zero for negative *F*^2^. The threshold expression of *F*^2^ > σ(*F*^2^) is used only for calculating *R*-factors(gt) *etc*. and is not relevant to the choice of reflections for refinement. *R*-factors based on *F*^2^ are statistically about twice as large as those based on *F*, and *R*- factors based on ALL data will be even larger.

### Fractional atomic coordinates and isotropic or equivalent isotropic displacement parameters (Å^2^)

**Table:**

	*x*	*y*	*z*	*U*_iso_*/*U*_eq_	
Cl1	1.32887 (4)	0.17773 (4)	1.09957 (2)	0.02303 (9)	
O1	1.13067 (10)	0.70996 (9)	0.86903 (6)	0.0151 (2)	
O2	0.98638 (10)	0.97666 (9)	0.74249 (6)	0.0174 (2)	
N1	0.91782 (13)	0.75551 (12)	0.76336 (7)	0.0151 (3)	
N2	0.80263 (13)	0.76074 (12)	0.70613 (7)	0.0146 (3)	
C1	1.30520 (15)	0.33360 (15)	1.04901 (8)	0.0159 (3)	
C2	1.17767 (16)	0.35872 (14)	1.00795 (8)	0.0168 (3)	
H2	1.1040	0.2921	1.0078	0.020*	
C3	1.15907 (16)	0.48241 (13)	0.96720 (8)	0.0159 (3)	
H3	1.0720	0.5002	0.9389	0.019*	
C4	1.26701 (15)	0.58168 (15)	0.96708 (9)	0.0149 (3)	
C5	1.39448 (15)	0.55382 (14)	1.00941 (8)	0.0176 (3)	
H5	1.4685	0.6201	1.0100	0.021*	
C6	1.41381 (15)	0.43007 (14)	1.05056 (9)	0.0175 (3)	
H6	1.5003	0.4117	1.0794	0.021*	
C7	1.24400 (15)	0.71056 (14)	0.92346 (8)	0.0153 (3)	
C8	1.30641 (15)	0.83596 (15)	0.92271 (8)	0.0175 (3)	
H8	1.3868	0.8635	0.9547	0.021*	
C9	1.22900 (14)	0.91848 (15)	0.86505 (9)	0.0165 (3)	
H9	1.2472	1.0113	0.8515	0.020*	
C10	1.12421 (14)	0.83819 (14)	0.83345 (8)	0.0137 (3)	
C11	1.00485 (15)	0.86322 (14)	0.77523 (8)	0.0138 (3)	
C12	0.66234 (15)	0.78352 (14)	0.74892 (9)	0.0163 (3)	
C13	0.65461 (17)	0.92221 (15)	0.79274 (9)	0.0240 (4)	
H13A	0.7283	0.9270	0.8360	0.036*	
H13B	0.5604	0.9333	0.8184	0.036*	
H13C	0.6701	0.9952	0.7518	0.036*	
C14	0.55159 (16)	0.77717 (16)	0.67895 (10)	0.0241 (4)	
H14A	0.5713	0.8491	0.6380	0.036*	
H14B	0.4561	0.7906	0.7025	0.036*	
H14C	0.5563	0.6877	0.6516	0.036*	
C15	0.63850 (16)	0.66767 (15)	0.81165 (9)	0.0230 (3)	
H15A	0.6501	0.5795	0.7834	0.035*	
H15B	0.5419	0.6742	0.8347	0.035*	
H15C	0.7083	0.6751	0.8570	0.035*	
H2A	0.8186 (15)	0.8364 (13)	0.6731 (8)	0.012 (4)*	
H1A	0.9484 (16)	0.6722 (16)	0.7743 (9)	0.029 (5)*	

### Atomic displacement parameters (Å^2^)

**Table:**

	*U*^11^	*U*^22^	*U*^33^	*U*^12^	*U*^13^	*U*^23^
Cl1	0.02355 (19)	0.02007 (19)	0.0255 (2)	0.00437 (17)	−0.00042 (17)	0.00723 (17)
O1	0.0162 (5)	0.0122 (5)	0.0169 (5)	0.0000 (4)	−0.0040 (4)	0.0010 (4)
O2	0.0205 (5)	0.0104 (5)	0.0214 (5)	−0.0001 (4)	−0.0019 (5)	0.0019 (4)
N1	0.0154 (6)	0.0107 (7)	0.0192 (7)	0.0012 (5)	−0.0047 (5)	0.0004 (5)
N2	0.0145 (7)	0.0146 (7)	0.0146 (6)	0.0003 (5)	−0.0032 (5)	0.0022 (5)
C1	0.0218 (8)	0.0154 (7)	0.0105 (7)	0.0048 (7)	0.0022 (6)	0.0016 (6)
C2	0.0168 (7)	0.0180 (8)	0.0157 (7)	−0.0019 (6)	0.0012 (7)	−0.0011 (6)
C3	0.0146 (7)	0.0187 (8)	0.0144 (7)	0.0016 (6)	−0.0031 (6)	−0.0010 (6)
C4	0.0157 (7)	0.0162 (8)	0.0128 (7)	0.0021 (6)	−0.0004 (6)	−0.0034 (6)
C5	0.0180 (8)	0.0172 (8)	0.0177 (8)	−0.0014 (6)	−0.0022 (6)	−0.0043 (6)
C6	0.0157 (8)	0.0225 (8)	0.0144 (8)	0.0045 (7)	−0.0029 (6)	−0.0021 (6)
C7	0.0125 (7)	0.0185 (8)	0.0150 (8)	0.0015 (6)	−0.0024 (6)	−0.0025 (6)
C8	0.0137 (7)	0.0185 (7)	0.0202 (8)	−0.0026 (6)	−0.0021 (6)	−0.0018 (6)
C9	0.0169 (7)	0.0129 (7)	0.0196 (8)	−0.0008 (6)	0.0022 (6)	0.0011 (6)
C10	0.0157 (7)	0.0106 (7)	0.0149 (7)	0.0023 (6)	0.0015 (6)	0.0004 (6)
C11	0.0139 (7)	0.0139 (8)	0.0137 (7)	0.0019 (6)	0.0041 (6)	−0.0023 (6)
C12	0.0134 (7)	0.0168 (7)	0.0187 (8)	0.0007 (6)	−0.0017 (7)	0.0000 (6)
C13	0.0207 (9)	0.0222 (8)	0.0292 (9)	0.0024 (7)	−0.0007 (7)	−0.0052 (7)
C14	0.0209 (8)	0.0245 (9)	0.0270 (9)	0.0008 (7)	−0.0075 (7)	0.0012 (7)
C15	0.0224 (8)	0.0230 (8)	0.0237 (8)	−0.0003 (8)	−0.0005 (7)	0.0029 (7)

### Geometric parameters (Å, °)

**Table:**

Cl1—C1	1.7411 (14)	C6—H6	0.9500
O1—C7	1.3744 (15)	C7—C8	1.360 (2)
O1—C10	1.3795 (16)	C8—C9	1.4252 (19)
O2—C11	1.2398 (15)	C8—H8	0.9500
N1—C11	1.3465 (17)	C9—C10	1.3561 (18)
N1—N2	1.4174 (16)	C9—H9	0.9500
N1—H1A	0.882 (15)	C10—C11	1.4771 (19)
N2—C12	1.4999 (18)	C12—C14	1.5289 (19)
N2—H2A	0.922 (13)	C12—C13	1.5296 (18)
C1—C2	1.3866 (19)	C12—C15	1.5311 (18)
C1—C6	1.3889 (19)	C13—H13A	0.9800
C2—C3	1.3861 (17)	C13—H13B	0.9800
C2—H2	0.9500	C13—H13C	0.9800
C3—C4	1.4029 (19)	C14—H14A	0.9800
C3—H3	0.9500	C14—H14B	0.9800
C4—C5	1.4009 (18)	C14—H14C	0.9800
C4—C7	1.4578 (19)	C15—H15A	0.9800
C5—C6	1.3906 (19)	C15—H15B	0.9800
C5—H5	0.9500	C15—H15C	0.9800
			
C7—O1—C10	106.97 (10)	C10—C9—H9	126.8
C11—N1—N2	121.66 (12)	C8—C9—H9	126.8
C11—N1—H1A	119.9 (10)	C9—C10—O1	109.95 (12)
N2—N1—H1A	114.2 (10)	C9—C10—C11	133.51 (13)
N1—N2—C12	112.24 (10)	O1—C10—C11	116.41 (12)
N1—N2—H2A	106.0 (9)	O2—C11—N1	123.82 (13)
C12—N2—H2A	106.6 (9)	O2—C11—C10	121.41 (13)
C2—C1—C6	121.36 (13)	N1—C11—C10	114.74 (12)
C2—C1—Cl1	119.06 (11)	N2—C12—C14	104.77 (11)
C6—C1—Cl1	119.58 (11)	N2—C12—C13	112.52 (12)
C3—C2—C1	119.11 (13)	C14—C12—C13	109.88 (12)
C3—C2—H2	120.4	N2—C12—C15	108.52 (11)
C1—C2—H2	120.4	C14—C12—C15	110.58 (12)
C2—C3—C4	120.97 (14)	C13—C12—C15	110.44 (12)
C2—C3—H3	119.5	C12—C13—H13A	109.5
C4—C3—H3	119.5	C12—C13—H13B	109.5
C5—C4—C3	118.69 (13)	H13A—C13—H13B	109.5
C5—C4—C7	121.77 (13)	C12—C13—H13C	109.5
C3—C4—C7	119.54 (13)	H13A—C13—H13C	109.5
C6—C5—C4	120.66 (13)	H13B—C13—H13C	109.5
C6—C5—H5	119.7	C12—C14—H14A	109.5
C4—C5—H5	119.7	C12—C14—H14B	109.5
C1—C6—C5	119.20 (13)	H14A—C14—H14B	109.5
C1—C6—H6	120.4	C12—C14—H14C	109.5
C5—C6—H6	120.4	H14A—C14—H14C	109.5
C8—C7—O1	109.33 (12)	H14B—C14—H14C	109.5
C8—C7—C4	136.18 (13)	C12—C15—H15A	109.5
O1—C7—C4	114.49 (12)	C12—C15—H15B	109.5
C7—C8—C9	107.33 (13)	H15A—C15—H15B	109.5
C7—C8—H8	126.3	C12—C15—H15C	109.5
C9—C8—H8	126.3	H15A—C15—H15C	109.5
C10—C9—C8	106.41 (13)	H15B—C15—H15C	109.5
			
C11—N1—N2—C12	−101.13 (14)	O1—C7—C8—C9	−0.05 (16)
C6—C1—C2—C3	0.5 (2)	C4—C7—C8—C9	−179.46 (15)
Cl1—C1—C2—C3	−179.57 (10)	C7—C8—C9—C10	−0.42 (16)
C1—C2—C3—C4	0.0 (2)	C8—C9—C10—O1	0.73 (16)
C2—C3—C4—C5	−0.3 (2)	C8—C9—C10—C11	176.25 (14)
C2—C3—C4—C7	179.88 (13)	C7—O1—C10—C9	−0.77 (15)
C3—C4—C5—C6	0.2 (2)	C7—O1—C10—C11	−177.14 (11)
C7—C4—C5—C6	180.00 (13)	N2—N1—C11—O2	5.2 (2)
C2—C1—C6—C5	−0.6 (2)	N2—N1—C11—C10	−176.72 (11)
Cl1—C1—C6—C5	179.45 (10)	C9—C10—C11—O2	1.9 (2)
C4—C5—C6—C1	0.3 (2)	O1—C10—C11—O2	177.18 (12)
C10—O1—C7—C8	0.49 (14)	C9—C10—C11—N1	−176.21 (15)
C10—O1—C7—C4	−179.95 (11)	O1—C10—C11—N1	−0.92 (17)
C5—C4—C7—C8	−15.6 (3)	N1—N2—C12—C14	−176.55 (11)
C3—C4—C7—C8	164.26 (16)	N1—N2—C12—C13	64.11 (15)
C5—C4—C7—O1	165.04 (12)	N1—N2—C12—C15	−58.41 (14)
C3—C4—C7—O1	−15.13 (19)		

### Hydrogen-bond geometry (Å, °)

**Table:**

*D*—H···*A*	*D*—H	H···*A*	*D*···*A*	*D*—H···*A*
N1—H1A···O2^i^	0.882 (15)	2.026 (16)	2.8744 (15)	160.9 (14)

Symmetry codes: (i) −*x*+2, *y*−1/2, −*z*+3/2.

## References

[bb1] Dhadialla, T. S., Carlson, G. R. & Le, D. P. (1998). *Annu. Rev. Entomol.* **43**, 545–569. 10.1146/annurev.ento.43.1.545 9444757

[bb2] Flack, H. D. (1983). *Acta Cryst.* A**39**, 876–881.

[bb3] Heller, J. J., Mattioda, H., Klein, E. & Sagenmuller, A. (1992). *Brighton Crop. Prot. Conf. Pests. Dis.* **1**, 59–66.

[bb4] Li, X. C., Yang, X. L., Kai, Z. P. & Ling, Y. (2006). *Huaxue Tongbao*, **69**, 668–673.

[bb5] Mao, C. H., Wang, Q. M., Huang, R. Q., Bi, F. C., Chen, L., Liu, Y. X. & Shang, J. (2004). *J. Agric. Food Chem.* **52**, 6737–6741. 10.1021/jf048834e 15506810

[bb6] Rigaku/MSC (2005). *CrystalClear* (Version 1.36) and *CrystalStructure* (Version 3.7.0). Rigaku/MSC, The Woodlands, Texas, USA.

[bb7] Sheldrick, G. M. (2008). *Acta Cryst.* A**64**, 112–122. 10.1107/S0108767307043930 18156677

[bb8] Wing, K. D. (1988). *Science*, **241**, 467–469. 10.1126/science.3393913 3393913

[bb9] Wing, K. D., Slawecki, R. A. & Carlson, G. R. (1988). *Science*, **241**, 470–472. 10.1126/science.241.4864.470 17792610

[bb10] Yang, X. L., Qian, J. H., Chen, F. H. & Wang, D. Q. (2002). CN Patent No. 1370405.

